# HIV control programs reduce HIV incidence but not HCV incidence among people who inject drugs in HaiPhong, Vietnam

**DOI:** 10.1038/s41598-020-63990-w

**Published:** 2020-04-24

**Authors:** Jean-Pierre Molès, Roselyne Vallo, Pham Minh Khue, Duong Thi Huong, Khuat Thi Hai Oanh, Nguyen Thi Thoa, Hoang Thi Giang, Nham Thi Tuyet Thanh, Vu Hai Vinh, Tuyet Anh Bui Thi, Marianne Peries, Kamyar Arasteh, Catherine Quillet, Jonathan Feelemyer, Laurent Michel, Don Des Jarlais, Didier Laureillard, Nicolas Nagot

**Affiliations:** 1 Pathogenesis and control of chronic infections, Inserm, Etablissement Français du Sang, University of Montpellier, Montpellier, France; 2 Hai Phong University of Medicine and Pharmacy, Faculty of Public Health, Hai Phong, Vietnam; 3 Supporting Community Development Initiatives, Hanoi, Vietnam; 4 Haiphong Provincial AIDS Center, Hai Phong, Vietnam; 5 Infectious Diseases Department, Viet Tiep Hospital, Hai Phong, Vietnam; 6 College of Global Public Health, New York University, New York, USA; 7 CESP/Inserm U1018, Pierre Nicole Centre, French Red Cross, Paris, France; 8 Infectious Diseases Department, Caremeau University Hospital, Nîmes, France

**Keywords:** Epidemiology, Risk factors

## Abstract

In Vietnam, harm reduction programs to control HIV among people who inject drugs (PWID) were implemented approximately 10 years ago. Since then, the HIV prevalence has declined in this population, however, the impact of these programs on the rate of new HIV and Hepatitis C (HCV) infections remains unknown as high mortality can exceed the rate of new infections. We evaluated HIV and HCV incidences in a cohort of active PWID in HaiPhong in 2014, who were recruited from a community-based respondent driven sampling (RDS) survey and followed for 1 year. Only HIV-negative or HCV-negative participants not on medication assisted treatment (MAT) were eligible. HIV/HCV serology was tested at enrollment and at 32- and 64-week follow-up visits. Among 603 RDS participants, 250 were enrolled in the cohort, including 199 HIV seronegative and 99 HCV seronegative PWID. No HIV seroconversion was reported during the 206 person-years (PY) of follow-up (HIV incidence of 0/100PY, one-sided 97.5%CI:0-1.8/100 PY). Eighteen HCV seroconversions were reported for an incidence of 19.4/100 PY (95%CI;11.5-30.7). In multivariate analysis, “Injecting more than twice daily” was associated with HCV seroconversion with an adjusted odds ratio of 5.8 (95%CI;1.8–18.1). In Hai Phong, in a context that demonstrates the effectiveness of HIV control programs, the HCV incidence remains high. New strategies such as mass access to HCV treatment should be evaluated in order to tackle HCV transmission among PWID.

## Introduction

People who inject drugs (PWID) are at increased risk of blood borne infections. Among these infections, HIV and Hepatitis C (HCV) infections remain highly endemic in this high-risk group. The latest meta-analysis by Degenhardt *et al*. estimated that there are 15.6 million PWID worldwide, with an HIV prevalence of 17.8% and an HCV prevalence of 52.3%. The authors mentioned that the quality of the data from low/middle income countries are disparate and scarce^1^.

In Vietnam, among the estimated 220,000 PWID in 2013, HIV prevalence ranged from 10 to 45% in different provinces^2^. Beginning in 2015, the country modified its legal and policy framework with support from international agencies (Global Fund to fight AIDS, tuberculosis and Malaria, President’s Emergency Plan for AIDS Relief) toward more evidence-based programs, including harm reduction through widespread needle and syringe provision (NSP), medication assisted treatment (MAT) and an increasing number of outpatient clinics for HIV care^3^. Both large scale access to sterile needles and syringes^4,5^ and MAT^6–8^ have proved efficacious in reducing HIV incidence. In most of Europe and in some North American settings such as New York City and British Columbia, these interventions, along with intensive HIV care, significantly reduced HIV transmission among PWID^9^.

While significant effort has been put forth to tackle HIV infection among PWID, the HCV epidemic has been comparatively neglected. Among PWID in Vietnam, HCV sero-prevalence (people who have been infected may clear the virus naturally but not the antibody response) ranged from 31% to 97% in different areas of the country in 2015^10–14^. In 2015; Clatts *et al*. confirmed the very active dynamics of HCV infection within PWID in Hanoi. They recruited 179 young male self-reporting heroin injectors and reported a HCV prevalence of 46% and an incidence of 23.4/100 person-years (PY) (95%CI: 11.65–41.78); however, there was only 47 PY of follow-up and 64% of participants were lost-to-follow up at 16 months, making these findings difficult to generalize^15^. The recent evaluation of harm reduction programs on HCV incidence concluded that MAT alone has a strong impact but it was unclear if NSP had the same impact^16^. For example, British Columbia showed a marked decline in HCV incidence from 25% in 1999 to 4.9% in 2012 but residual HCV transmission remained unacceptably high^17^.

Hai Phong is a city of 2 million inhabitants with about 10,000 PWID, 13 methadone clinics and 12 outpatient HIV clinics in operation in 2014. Syringes and needles are available mainly in private pharmacies, but also through peer-groups. Hai Phong was chosen as a model city to evaluate whether programmatic interventions which proved successful for eliminating HIV transmission among PWID in high income countries could be adapted to low/middle income countries.

In order to assess the feasibility of implementing such a research program, in 2014, we conducted a respondent driven sampling (RDS) study among active heroin injectors^18^. In this context, we reported that both the HCV-antibody prevalence (66%) and HIV prevalence (25%) were high^18^. However, it was important to estimate the current rate of new HIV and HCV infections among PWID, and to identify the PWID profiles and behaviors associated with new infections in order to gain insight on the local routes of transmission and tailor prevention activities based on these behaviors.

## Results

### Study population characteristics

**Figure 1:**
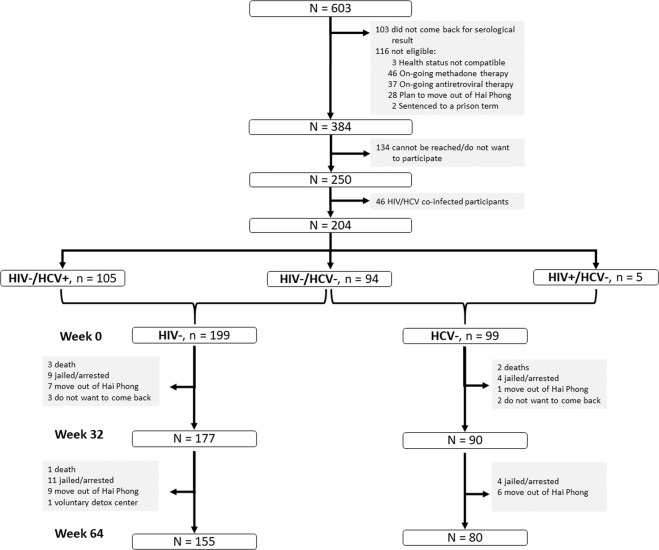
Study flow chart.

Out of the 603 RDS participants who reported using injection drugs, had recent skin marks of injection and had an injectable drug detected in urine, 384 met the cohort inclusion criteria (not being on MAT and having a life compatible with the study visit schedule, for more details see M&M section) and 250 were recruited (Fig. 1). All were injecting heroin; only 1 was injecting methamphetamine. Among the 250 cohort participants, 204 PWID had HIV negative and/or HCV negative serology at baseline and were used to assemble the HIV negative group (n = 199) and the HCV negative group (n = 99) for follow-up. Their characteristics are presented in Table 1. Overall, the two groups presented very similar characteristics with a median age of 36 years and 5–6 years of injection history. The majority of the participants injected more than 2 times a day, and a quarter of them had smoked methamphetamine in the previous 3 months. They reported low rates of needle and syringe sharing.

**Table 1:** Baseline characteristics among PWID cohort participant in Hai Phong.

	HIV negative n = 199	HCV negative n = 99
**Sex** (Male)	179(89.9%)	89(89.9%)
**Age** (median [IQR])	37[30–45]	36[30–43]
**School**		
Never been to school	2(1.0%)*	0(0%)
Grade 1 to 5	20(10.1%)	7(7.1%)
Grade 6 to 9	93(47.2%)	43(43.4%)
Grade 10 to 12	73(37.1%)	42(42.4%)
Above grade 12	9(4.6%)	7(7.1%)
**Marital status**		
Single	61(30.6%)	25(25.3%)
Legally married or had wedding	70(35.2%)	38(38.4%)
Living couple without marriage	4(2.0%)	1(1.0%)
Divorced/separated	60(30.2%)	34(34.3%)
Widowed	4(2.0%)	1(1.0%)
Having a medical insurance at RDS	35(17.6%)	17(17.2%)
Having ever been arrested	127(63.8%)	63(63.6%)
Number of years of injection^¥^	6[2–12]	5[1–9]
**Age at first injection**		
<18 years old	7(3.5%)	0(0.0%)
18–29	95(47.7%)	43(43.4%)
30–39	60(30.2%)	39(39.4%)
>=40 years old	37(18.6%)	17(17.2%)
Number of injections during the last month	90[60–90]	78[60–90]
**Use of non-injecting drug in the last 3 months**		
- heroin	6(3.0%)	5(5.1%)
- ecstasy	4(2.0%)	3(3.0%)
- ketamine	3(1.5%)	0(0%)
- methamphetamine	56(28.1%)	27(27.3%)
Injection with needle/syringe used by some else	7(3.5%)	2(2.0%)
Number of sexual partners in the last 3 months	1[1–4]^‡^	1[1–4]^†^
Having at-risk sexual behaviors with the casual partner (no use of condom)	4(2.0%)	1(1.0%)
Men who have sex with men (last 3 months)	19(9.6%)	12(12.1%)
Female commercial sex work (last 3 months)	17(8.5%)	9(9.1%)
HIV-positive serology		5(5.1%)
HCV-positive serology	105(52.8%)	
Detectable HCV viral load among HCV-positive serology	86(82.7%)^¥^	
Median HCV viral load (cp/ml)	158,500[27,100–663,000]	

Data are median (IQR), or %(n). *2 missing values, ¥1 missing value, ‡n = 103; †n = 55. Note that all but 5 of the HCV seronegatives were also HIV seronegative and contributed to both the HIV and HCV incidence analyses.

Of note, the participants enrolled in these follow-up studies had similar characteristics to the RDS participants who were not enrolled (n = 399), with the exception of age (median: 37 years [IQR: 30;44] vs 35[30;41] respectively, p = 0.02), age at first injection (median: 29 years [Interquartile range (IQR): 24;37] vs 24 years [IQR: 21;31] respectively, p < 0.001) and having a child (52% vs 65%, p = 0.004) (Table 2).

**Table 2:** Comparison of the characteristics of the PWID enrolled in the HIV and HCV seronegative cohorts versus those not enrolled in the cohorts.

	Follow up cohorts Baseline N = 204	Population not selected N = 399	P value (Chi square test or median test)
Sex: Male	183(90%)	359(90%)	0.9
Age	37[30–44]	35[30–41]	0.02
School:<grade 9 vs >	119(59%)	226(57%)	0.6
Marital status: legally married or in couple	75(37%)	128(32%)	0.3
Having a medical insurance	37(18%)	68(17%)	0.7
having a child	132(65%)	209(52%)	0.004
Age at first injection	29[24–37]	24[21–31]	>0.001

### Cohort follow-up

The retention rate after 64 weeks in the HIV-negative and the HCV-negative cohorts were 78% and 81%, giving a total of 212 PY and 108 PY, respectively (Fig. 1). Overall, 715 visits were completed out of the 816 visits planned (88%). The main reasons for missing data/loss to follow up among the study participants were similar in the two cohorts: having been sentenced to a prison term (n = 20 and n = 8, respectively), having moved out of Hai Phong (n = 16 and n = 7, respectively) and death (n = 4 and n = 2, respectively, including 2 suicides and 1 overdose). Only 3 participants withdrew from the study, one from the HIV-negative cohort and 2 from the HCV-negative cohort.

### HIV and HCV incidence

No participant seroconverted for HIV during the follow-up period, giving an estimated HIV incidence ranging between 0 and 1.8/100PY (97.5% one-sided CI).

Eighteen HCV seroconversions were detected during follow-up, including 13 during the first eight months, giving an overall HCV incidence of 19.4/100PY (95%CI: 11.5–30.7). HCV incidence was similar among Female Sex Worker (FSW)/PWID and Men having Sex with Men (MSM)/PWID and the other PWID (22.3/100PY (95%CI: 2.7–80.4), 22.0/100PY (95%:4.5–64.4), 18.6/100PY (95%CI: 9.9–31.8) respectively).

### Risk factors for HCV seroconversion

In bivariate analysis (Table 3), factors inversely associated with HCV seroconversion included “Use of methamphetamine” and “Use of non-injectable drugs other than methamphetamine.” Factors directly associated with HCV seroconversion were “Injecting more 60 times per month”, “Having a primary sexual partner who previously injected drugs”, and “Having been arrested at least once during the at-risk period”. These five variables were included as possible confounders in the multivariate model (Table 3) and the variable “Number of injections per month”, i.e. participants who injecting more than twice a day versus the participants who injected less than twice a day, remained positively associated with HCV seroconversion with an adjusted odds ratio of 5.8 (95%:1.8;18.1).

**Table 3:** Factors associated with HCV seroconversion among PWID in Hai Phong.

	Number of participants in each category		Bivariate model Crude OR (95%CI)	P value
	No seroconversion n = 74	Seroconversion n = 18		
Sex* (female vs male/transgender)	7(9.5%)	3(16.7%)	1.9(0.3–9.6)	0.40
Age*				
<32 years vs > =40	23(31.1%)	6(33.3%)	1.0(0.2–3.7)	0.89
[32;40] vs > =40	21(28.4%)	4(22.2%)	0.7(0.1–3.1)	
Having a low school grade (<grade 9)^§^	39(52.7%)	10(55.6%)	1.1(0.4–3.2)	0.83
Being in couple/married^§^	31(41.9%)	6(33.3%)	0.7(0.2–2.0)	0.51
Having a medical insurance*	12(16.2%)	4(22.2%)	1.5(0.3–5.9)	0.73
Younger injector (duration of injection ≤2 years)^§^	23(31.1%)	8(44.4%)	1.8 (0.6–5.1)	0.29
Inject > = 60 per month^§^	23(31.1%)	13(72.2%)	**5.9(1.8–18.1)**	0.003
Use of methamphetamine^§^	47(63.5%)	8(44.4%)	**0.5(0.2–1.3)**	0.14
Use of non-injectable drugs other than methamphetamine*	23(31.1%)	2(11.1%)	**0.3(0.0–1.3**)	0.14
Injection with syringes already used by someone else*	1(1.4%)	1(5.6%)	4.2(0.0–341.5)	0.35
Having shared syringe already used by the participant*	12(16.2%)	2(11.1%)	0.6(0.1–3.4)	0.73
Having sexual intercourse^§^	53(71.6%)	12(66.7%)	0.8(0.3–2.4)	0.68
Sexual activity with the primary partner vs no sexual activity^§^	49(66.2%)	10(55.6%)	0.6(0.2–1.8)	0.40
Having a primary sexual partner who previously injected drugs*	3(4.1%)	2(13.3%)	**3.5(0.3–34.0)**	0.20
Sexual intercourse with sex workers*	6(8.1%)	3(16.7%)	2.2(0.3–12.0)	0.37
Sex work of the participant (male or female)*	10(13.5%)	2(11.1%)	0.8(0.1–4.3)	1.00
Have been arrested at least once during the at-risk period*	3(4.1%)	3(16.7%)	**4.6(0.6–38.0)**	0.09
Being in methadone treatment during all the at-risk period*	3(4.1%)	2(11.1%)	2.9(0.2–27.7)	0.25

§ normal model; * exact model; for exact logistic regression we used exact p value and for standard logistic regression we used p value of chi square test. Value in bold were included in the final model.

Of note, there was a relatively high odd ratio for “Having been arrested during the at-risk period” with HCV incidence in bivariate analysis (OR = 4.6). This association was not significant in either univariate or multivariable analysis but the power was low due to small numbers (n = 6 subjects arrested).

## Discussion

This observational cohort study provides an in-depth studies of HIV and HCV incidence rates among active PWID in Hai Phong, Vietnam. We implemented a unique cohort of high-risk and difficult-to-reach PWID not engaged in care, and achieved a very high rate of follow-up (80%), contrasting with most similar reports in the region^15^. The participants of this cohort were active PWID who had not experience a stay in closed settings during the follow-up. We report a low HIV incidence (between 0 and 1.8/100 PY) contrasting with a very high rate of new HCV seroconversions (19.4/100 PY). The difference between HIV and HCV incidence, while both HIV-negative and HCV-negative participants received harm reduction programs delivered by trained and experienced peers, raises questions on the injection practices which allow HCV but not HIV infection (particularly given the highly efficient nature of blood borne HCV transmission).

Hai Phong has consistently reported the highest HIV prevalence in Vietnam but a steady decrease has been observed since 2006^2^. The low current HIV incidence among PWID confirms that the HIV epidemic among PWID is declining. The latest HIV incidence study in the country was done between 2005 and 2007 in another province, Thai Nguyen, at a time when no dedicated HIV control program had been implemented. That study reported an incidence rate of 5.2/100 PY (95%CI: 3.5–7.6) in a similarly active PWID population^19^. All in all, HIV circulate but at a low level among PWID, likely as a result of (i) a successful harm reduction programs (access to methadone, needles, syringes, counselling by peers and community-based organizations), supported by the low frequency of syringe sharing reported in this cohort (Table 1) and by a recent qualitative study^20^; and (ii) the access to universal ART which started in 2010; our data showed that 60% of the HIV positive PWID were under treatment in 2015. With the estimated HIV prevalence was 25% in 2015, the risk of transmission persists in the PWID community through HIV-infected individuals not taking or failing on ART and the remaining sharing practice.

The HCV prevalence in Vietnam and within PWID populations in Vietnam has been described in various reports^14,21–23^. In Hai Phong, Ishizaki *et al*. reported a HCV prevalence varying from 62.1% in 2007, to 42.7% in 2008, and to 58.4% in 2012 among PWID recruited in drug rehabilitation center and not treated for HBV, HCV and HIV^24^. A single study reported an HCV incidence of 23.3/100PY (95%CI: 11.65–41.78) in 2006, similar to the HCV incidence documented for our study^12^. At that time, harm reduction programs were about to start but 10 years later, HCV incidence and prevalence remained the same in Hai Phong although the context evolved towards much safer injection practices. After adjusting for confounders in multivariable analysis, only the frequency of injection (above 2 injections per day) was significantly and strongly associated with HCV seroconversion, suggesting the risk is related to injection practices. The risk of transmission through injection equipment is limited due to minimal paraphernalia (syringe and sterile water solution or injectable anesthetic solution -Novocain solution is also used locally for this purpose) used to prepare the injecting drug solution. Indeed, the purity of heroin in Vietnam is very high and does not require heating and filtering before using, but simply diluting with sterile water before a straight venous injection. However, the knowledge of any viral transmission risk through dirty diluent is uncommon within this PWID community^25,26^ and many PWID reuse their syringe after rinsing which can contaminate the leftover diluent. Novocain solution sharing could likely represent an additional route of transmission of HCV but this hypothesis requires further investigation. Other known factors such as “Injection with syringes already used by someone else” were not associated with HCV seroconversion, but this could be due to under reporting of this behavior by PWID as a result of social desirability bias^25^. As noted above, we cannot exclude that police arrest was a risk factor for HCV, independent of the frequency of sharing. The frequent syringe sharing in prison might be an explanation, in Vietnam as in other places^27^. Of note, the current model did not include the variable “initiating MAT” because this variable is linked to the variable “number of injection per month”. It is noteworthy that if we interchanged the variables, the OR for “initiating MAT”in the new model is of 6.3 (1.8–29.1), p = 0.001. The identification of the main cause of infection is important to tailor peer-led education activities for both primary and secondary prevention. When the new antiviral HCV treatments become available in Vietnam, reducing the reinfection rate after cure will be a priority in areas with high background incidence^28^. Without a combined efficacious harm reduction and therapeutic interventions, the reinfection rate could still be high in such a high incidence context^29^, as already observed in large cohort from British Columbia in 2018^30^.

Taken together, we reported herein different rates of transmission of HIV and HCV. There are several possible reasons for this. At first, it is well known that a syringe contaminated with HCV has a 5 to 20 higher infectivity than the one contaminated with HIV^31^. Another explanation may result from the adoption of safer injection practices after the disclosure of HIV seropositivity. This hypothesis was supported by peers’ declarations substantiating that HIV-positive PWID never share their syringes. To date, the impact of needle exchange programs on HCV transmission among PWID remains unclear; a recent meta-analysis by Platt *et al*. reported a protective association between NSP and HCV incidence in Europe (relative risk (RR): 0.24 (0.09–0.62)) but not in North America (RR: 1.25 (0.63–2.46))^16^. Our data are more similar to those reported in North America. They also reported a clear protective effect of MAT on HCV incidence (RR: 0.51 (0.40–0.63) but our study cannot evaluate this association as too few of the participants were on MAT.

This study has several limitations. The representativeness of the cohort is limited because the main objective of the study was to demonstrate the feasibility of an interventional study among PWID in Hai Phong city rather than studying a representative sample of PWID. More precisely, the study population did not include PWID who experienced a stay in closed settings such as rehabilitation centers or prison. Indeed, the proportion of loss to follow-up related to these situations represented half of the total loss to follow-up. The current duration of incarceration are superior to 1 year, which make impossible to recapture them as per the design of the study. Sensitive analysis were not include to overcome this bias because data on HIV transmission in Vietnamese close setting are not available. The conclusion we draw from the observations reported herein applied to PWID not being incarcerated and not to the general PWID population of this city. Furthermore, the cohort sampling overrepresented populations that are even more hard-to-reach since they were not engaged in methadone or HIV care. By doing so, we diverge from the standard RDS procedures and consequently we did not apply RDS adjustments to our results. Indeed, they tended to be older and had recently started to inject, but their other characteristics were similar as those from the RDS participants. Enrolling older participants may underestimate the actual incidence values. The circulation of HCV within the PWID population is evaluated only among sero-negative participants, and cannot account for new infections among participants already seropositive. Reporting only seroconversion also underestimate the actual HCV incidence value. Risk factors analysis for HCV seroconversion was limited and further investigations are required to decipher other HCV risk factors (e.g. sharing of diluents, tattoos, piercing, smoking pipes). Finally, HCV seroconversion could take place up to 6 months after infection, but this delay varies between individual and it is difficult to document without nucleic acid testing.

In Hai Phong where harm reduction and access to ART programs have been implemented for 10 years, HIV transmission is likely controlled but transmission of HCV is not. There is now a clear need to implement an HCV screening program, to tailor harm reduction programs with a focus on HCV risk, and to develop large access to new HCV treatments has become a priority to control the HCV epidemic among PWID^32,33^.

## Methods

### Study design

We conducted an observational cohort study, with follow-up visits at week 12, 20, 32 and 64 (NCT02573948) in accordance with local and international guidelines. The implementation of this cohort and its follow-up were done with the direct contribution of local peer support groups of drug users. The study was reviewed and approved by the Institutional Review Boards of the Hai Phong University of Medicine and Pharmacy (Vietnam) and the Icahn School of Medicine at Mount Sinai (USA).

### Study population

We first recruited ‘active’ PWID using a RDS survey in Hai Phong, Vietnam, with the following inclusion criteria: reporting using injection drugs and having recent skin marks of injection and having an injectable drug detected in urine (heroin or methamphetamine). Details of the RDS phase have been described elsewhere^18^. Briefly, 12 seeds selected by the peers, received 3 coupons. After participating in the study themselves, they were asked to distribute the coupons to other PWID who would meet the inclusion criteria and participate in the study. Participants received an incentive when enrolled in the study and when they successfully recruited new participants. In three weeks, the study recruited 603 PWID. From the RDS survey, we recruited potential participants for a 1-year cohort with the following criteria: (i) not being on methadone maintenance therapy, (ii) having health status compatible with study visit schedule, (iii) not planning to move out of Hai Phong over the next two years, (iv) not being sentenced to an upcoming prison term and (v) having signed in informed consent form. Through these inclusion criteria, we wished to target PWID not already engaged in care (either MAT or ART) with a view to describe the HIV and HCV incidence among those most at risk, and to estimate the ability to engage these participants in care (MAT and/or ART) through peer support, as a pilot of a larger intervention.

Among the RDS participants meeting the above criteria, those being HIV-infected, having an HCV-negative serology and belonging to the hardest-to-reach groups i.e. men having sex with men (MSM), female sex worker (FSW) and early injectors (EI) were initially prioritized. The cohort was next randomly completed from the remaining eligible participants until we reached 250 participants. A specific consent form was signed by these participants. In the present work, we consider only HIV-negative and/or HCV-negative serology PWID enrolled in the cohort (Fig. 1). The cohort study started 7 weeks after the end of the RDS, baseline characteristics were those of the RDS.

### Study site and follow-up visits

The study took place in the office of a major peer support group in Hai Phong. PWID were individually followed by peers who scheduled the visits, called the participants for their follow-up visit on the day before the visit, and called them as well the day after a missed appointment. Home visits were conducted when appropriate.

At each visit in the study site, a standardized questionnaire was administered by a trained interviewer. This questionnaire addressed issues on participant’s drug use, sexual behaviors, medical and administration-related events (arrests, etc.). At baseline, weeks 32 and 64, drug tests were administered to detect drugs in urine (Drug-screen Multi 7 A card, Nal von Minden, Moers, Germany). HIV and HCV serology were repeated at the same visit. HIV testing using Determine™ HIV-1/2 (Alere™, Waltham, USA) rapid test. HIV confirmation was done using Bioline HIV1/2 3.0 rapid test (Standard Diagnostics Inc., Gyeonggi-do, Republic of Korea) plus the MUREX HIV Ag/Ab Combination test (Diasorin, Saluggia, Italy). HCV serology using either SD HCV ELISA 3.0 or SD Bioline HCV rapid test (Standard Diagnostics Inc., Gyeonggi-do, Republic of Korea). Plasma HCV RNA was quantitated after nucleic acid extraction using the ExiPrep™ Dx Viral DNA/RNA kit and HCV amplification using the AccuPower^®^ HCV Quantitative RT-PCR Kit on the ExiStation™ Molecular Diagnostic System (Bioneer Corporation, Daejeon, Republic of Korea).

During the cohort study, the participants benefited from various activities including free registration to the national health insurance system, peer support to facilitate access to the national medication assisted (methadone) treatment, antiretroviral therapy for HIV-positive participants, HIV/HCV counseling and access to HBV vaccination. They received 150,000 Vietnamese dong (US$ 7.50) at each visit as compensation for their time and travel costs.

### Data collection and data analyses

The data were collected through electronic case report forms on tablet computers.

Baseline participant characteristics were presented using number and percentage for categorical data; and median and interquartile range (IQR) for quantitative variables.

For HIV and HCV incidence estimation, we assumed that the infection occurred at midpoint between the last negative test and the first positive test. The incidence was then defined by the total number of new infections that occurred between the RDS and the final visit, divided by the quantity of follow-up (in person-years) during the at-risk period. Only participants with two test results were included in these analysis. They could have a follow-up time quantity of 32-weeks if lost to follow-up after the second visit or a time of 64-weeks if they completed the 64-week follow-up, whether they attended or did not the 32-week visit. We expressed the result with a two-sided 95% Poisson confidence interval (CI) for HCV, and one-sided 97.5% Poisson CI for HIV incidence. All the remaining statistical tests were two-tailed with alpha set at 5%.

The use of methadone or methamphetamine was determined by either a positive urine test or self-report in the interview.

All explanatory variables repeated over time through intermediate visits (W12, W20) were summarized in order to have one value for the at-risk period. For categorical variables, the ‘most-at-risk’ value was attributed to the considered follow-up period. For the quantitative variable such as age, we have created three categories using tercile range; for the number of injections per month, we created two categories.

We analyzed the risk factors for HCV seroconversion using classic and exact logistic regression models. All summarized variables associated with HCV seroconversion with a p value < 0.25 in bivariate analysis were considered as potential confounders and were included in the multivariable model. We used backward selection of the variables.

All statistical analyses were done with SAS software (Version 9.4 for Windows; Copyright© [2016] SAS Institute Inc.) and STATA software (version 11 for Windows; Copyright© [1984–2009]StataCorp).

## Data Availability

The datasets generated during and/or analysed during the current study are available from the corresponding author on reasonable request. Because the data include very sensitive health information, e.g., HIV status; however the data will be de-identified.
